# Supplementary material for: Nanostructured Polymethylsiloxane/Fumed Silica Blends
Source: Materials (Basel). 2019 Jul 28;12(15):2409. doi: 10.3390/ma12152409 (PMC6695803; doi:10.3390/ma12152409)
Supplement: Supplementary file 1 [file materials-12-02409-s001.pdf]

materials

Supplementary Materials

Nanostructured Polymethylsiloxane/Fumed Silica Blends

Iryna Protsak [1,2], Volodymyr M. Gun'ko [3], Volodymyr V. Turov [3], Tetyana V. Krupska [3], Eugeniy M. Pakhlov [3], Dong Zhang [4], Wen Dong [1,*], Zichun Le [2]

[1] College of Environment, Zhejiang University of Technology, Hangzhou 310014, China
[2] College of Science, Zhejiang University of Technology, Hangzhou 310023, China
[3] Chuiko Institute of Surface Chemistry of National Academy of Sciences of Ukraine, Kyiv 03164, Ukraine
[4] Department of Chemical & Biomolecular Engineering, University of Akron, Akron, OH 44325, USA
* Correspondence: dongwen@zjut.edu.cn

Received: 27 June 2019; Accepted: 26 July 2019; Published: 28 July 2019

TEM

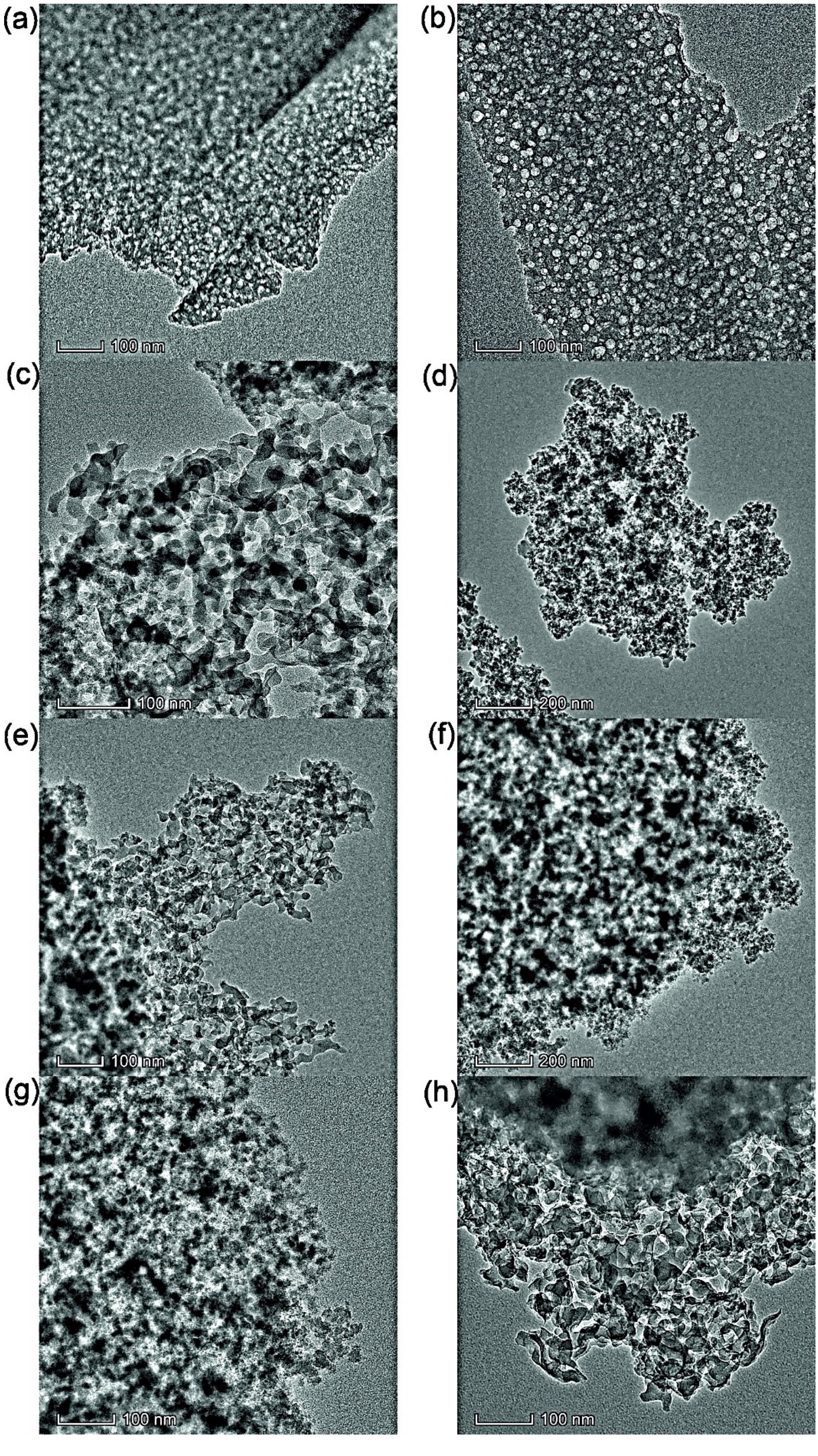

Figure S1. TEM images of (a) Bl, (**b**) Bh1l, (**c**) Bh1s, (**d**) Bh2l, (**e**) Bh2s, (**f**) Adl, (**g**) Pdl, and (**h**) Phdl (scale bar 100 nm (**a**-**d**, **h**) and 200 nm (**e**, **f**)).

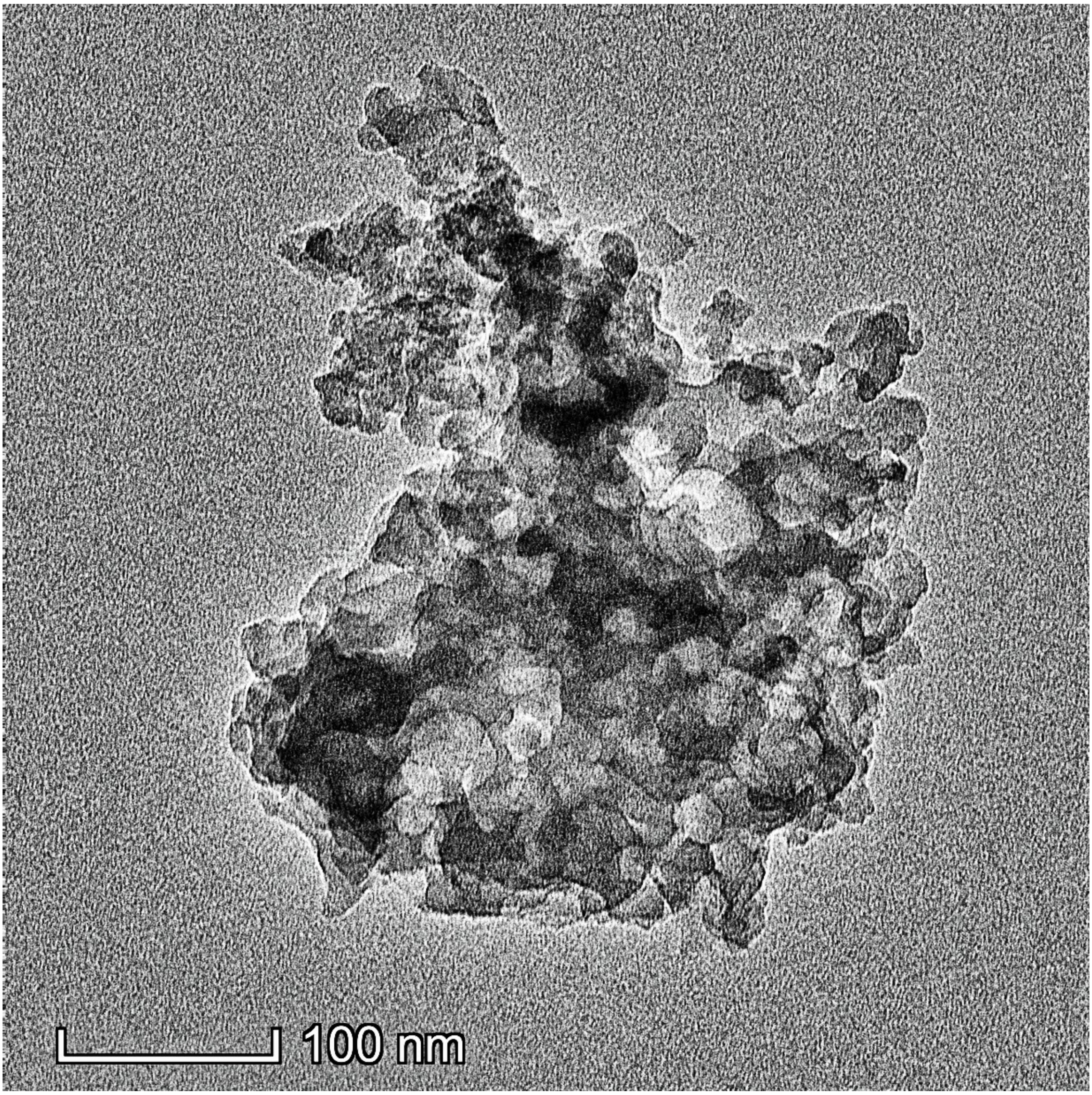

Figure S2. TEM image of Bh2l.

SEM

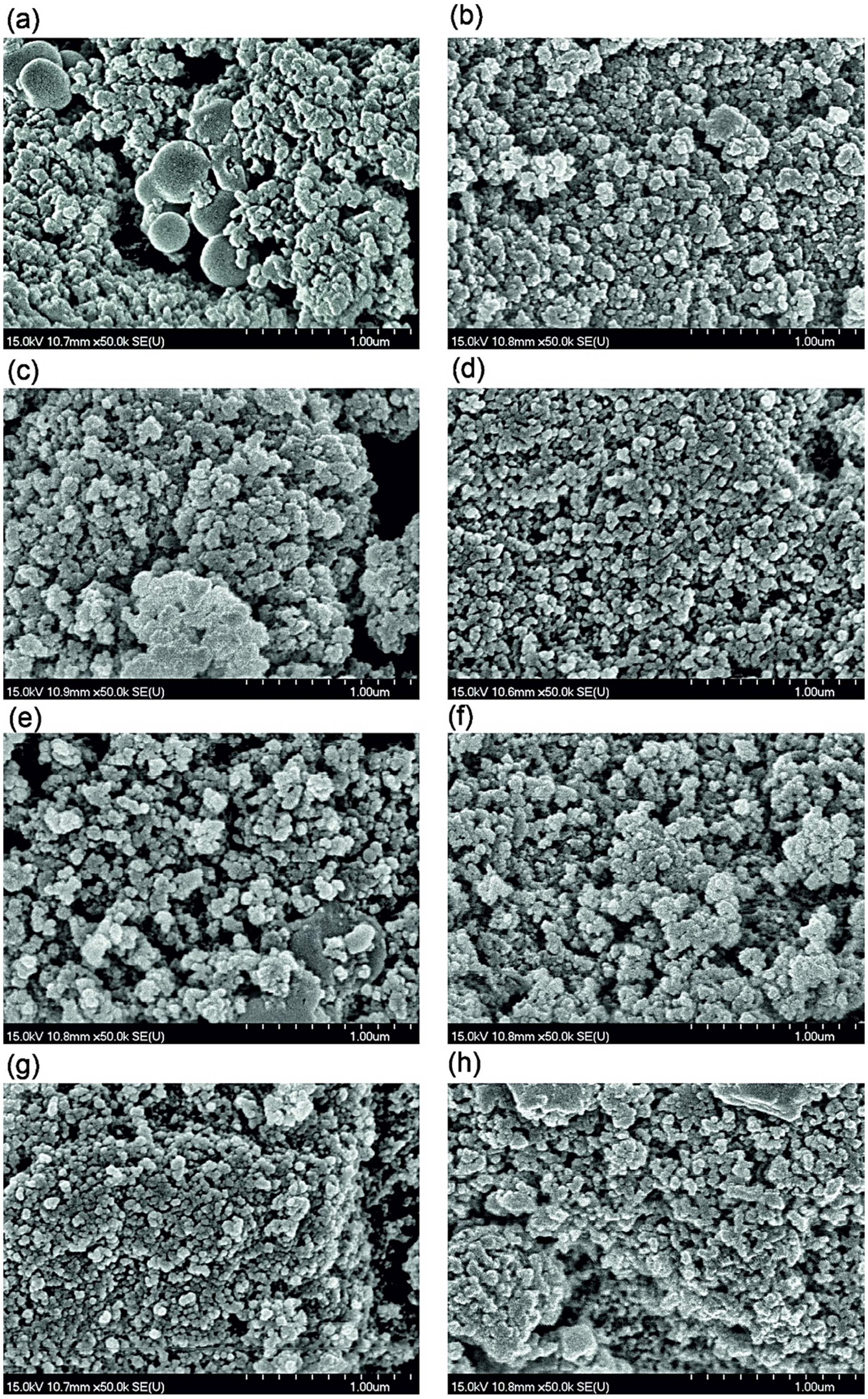

Figure S3. SEM images of Bl, (b) Bh1l, (c) Bh1s, (d) Bh2l, (e) Bh2s, (f) Adl, (g) Pdl, and (h) Phdl (scale bar 1 μm).

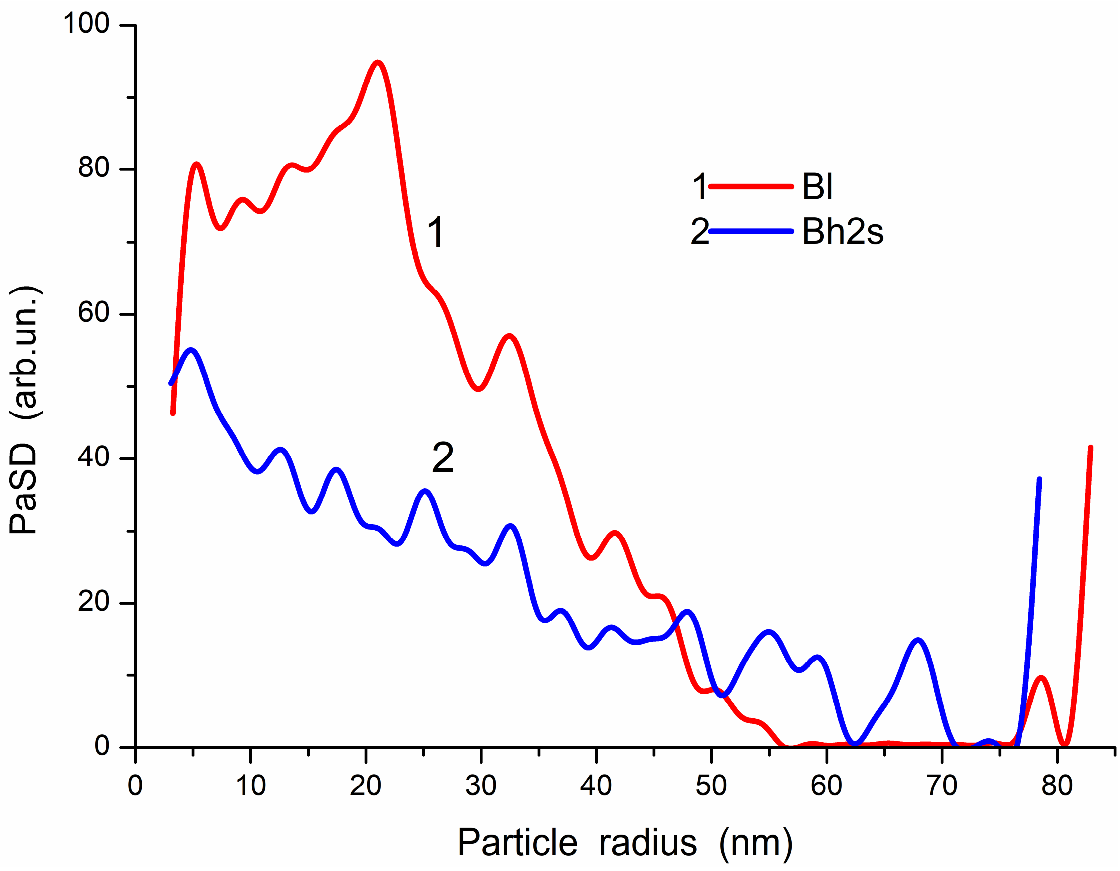

Figure S4. Particle size distributions (PaSD) of samples 1 (Bl) and 5 (Bh2s) calculated using SEM images (Figure S3) and Fiji software with local thickness plugin (https://imagej.net/Fiji).

Nitrogen adsorption

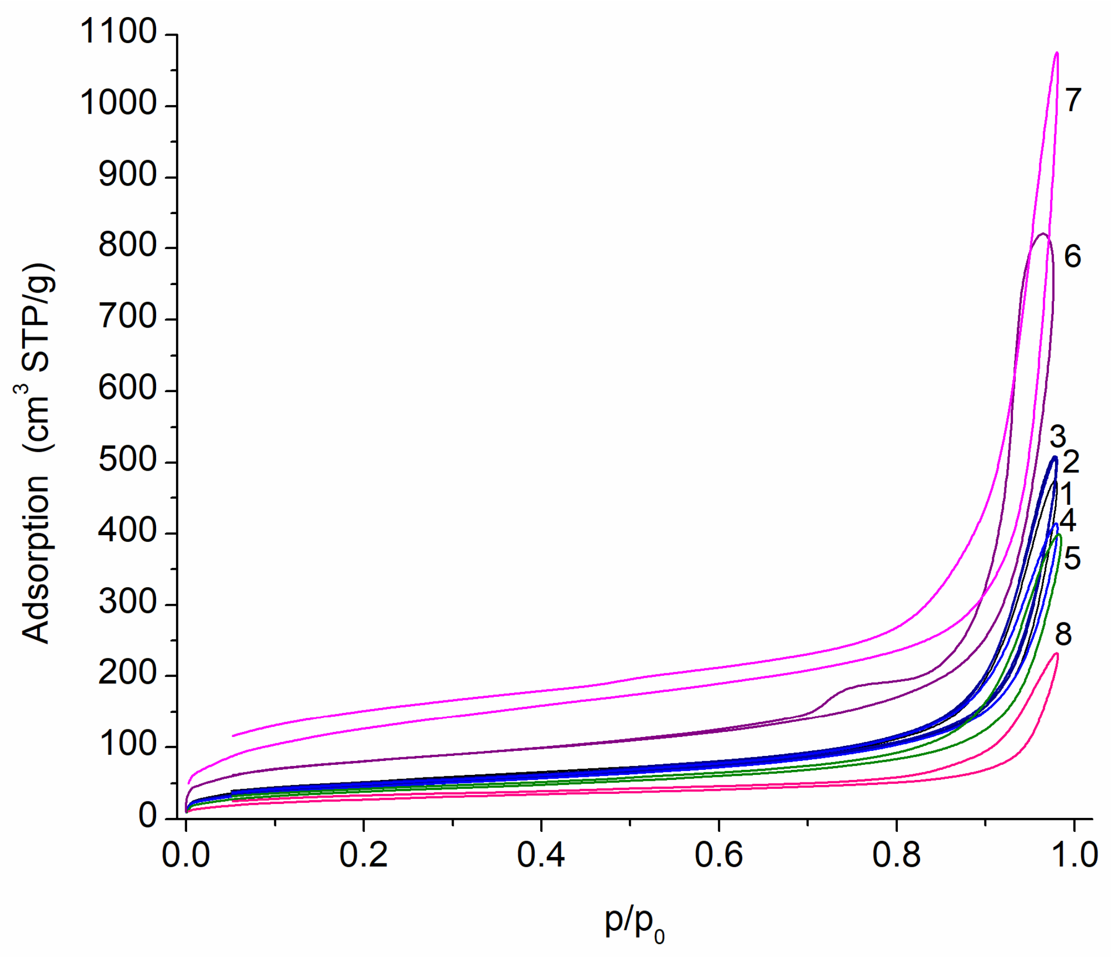

Figure S5. Nitrogen adsorption-desorption isotherms (77.4 K) for PMS, cA-300, and their blends (curve numbers correspond to sample numbers of in Table S1).

Differential NLDFT PSD

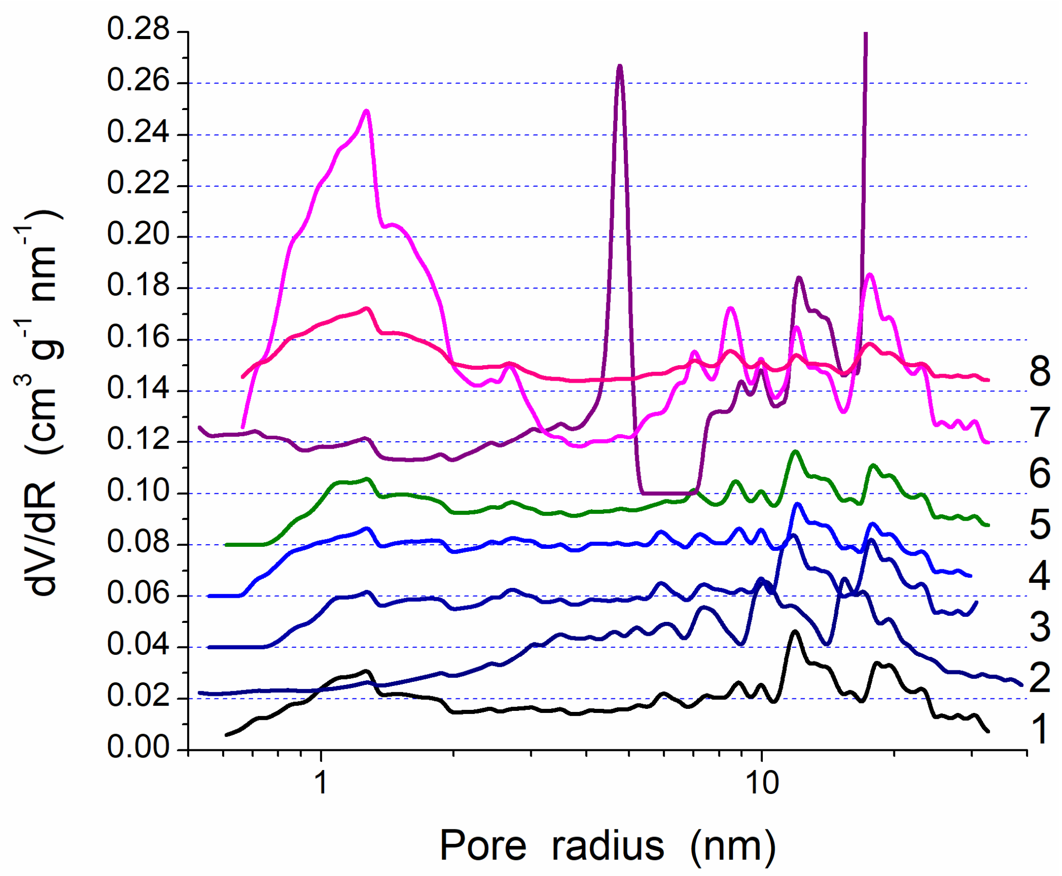

Figure S6. NLDFT PSD (equilibrium model with cylindrical pores in silica) for PMS, cA-300, and their blends (curve numbers correspond to sample numbers of Table S1).

^{29}Si CP/MAS NMR spectroscopy

Table S1. Contributions of various structures in PMS, cA-300, and their blends.

No	Sample composition	Sample label	Hydration (g/g)	T_2 (%)	T_3 (%)	Q_2 (%)	Q_3 (%)	Q_4 (%)
1	dPMS/cA-300	Bl	0.007	19.2	67.8	2.4	4.5	6.1
2	dPMS/cA-300	Bh1l	0.1	17.5	82.5	-	-	-
3	dPMS/cA-300	Bh1s	0.1	20.8	70.7	2.2	3.5	2.8
4	dPMS/cA-300	Bh2l	0.2	16.7	70.8	4.8	3.0	4.7
5	dPMS/cA-300	Bh2s	0.2	22.8	75.2	0.4	1.1	0.5
6	cA-300	Adl	0.008	-	-	25.1	56.2	18.7
7	dPMS	Pdl	0.004	12.1	87.9	-	-	-
8	PMS initial	Phdl	0.007	14.2	85.8	-	-	-

Note. Q_n corresponds to $Si(OH)_{4-n}(OSi\equiv)_n$ at $n = 2$ (−91~−93 ppm), 3 (−99~−102 ppm), 4 (−109~−111 ppm); T_3 (−70 ppm) corresponds to $(\equiv SiO)_3SiCH_3$ and T_2 (−60 ppm) corresponds to $(\equiv SiO)_2Si(OH)CH_3$.

IR spectroscopy

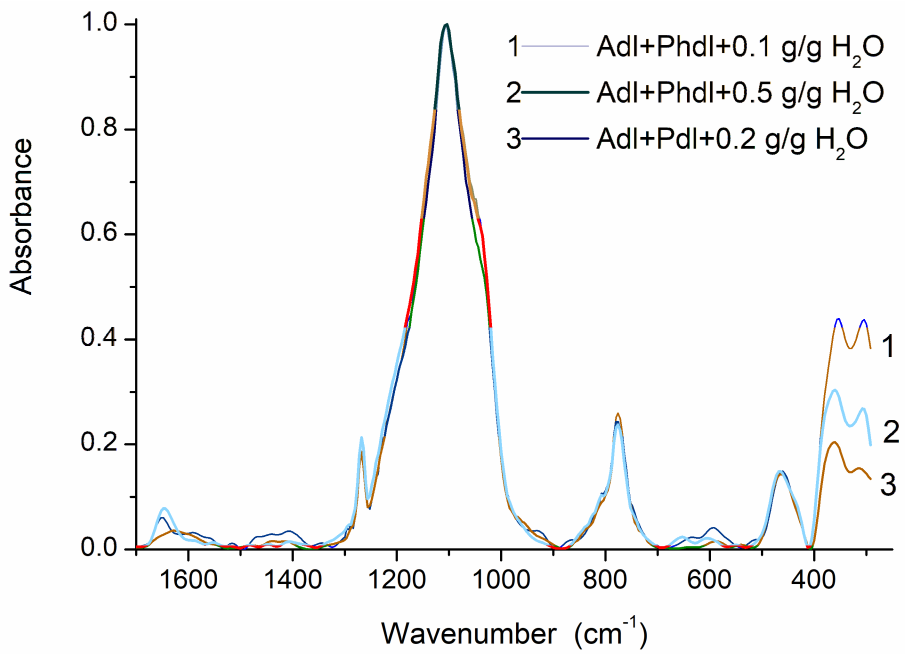

Figure S7. IR spectra (in the range of 1700–300 cm^{-1}) of dPMS/cA-300 blends differently hydrated before strong mechanical treatment (thin pallets were pressed using samples stirred with KBr as 1:400).

Quantum chemical calculations

The δ_H values for water clusters (up to $100H_2O$) were calculated as the difference in the isotropic values of the magnetic shielding tensors of H atoms ($\sigma_{H,iso}$) of tetramethylsilane, TMS ($\delta_{H,TMS} = 0$ ppm) as a reference compound (*e.g.*, $\sigma_{H,iso}$ = 31.76 and 31.40 ppm for tetramethylsilane (TMS) by GIAO/B3LYP/6-31G(d,p) and GIAO/ωB97X–D/cc–pVDZ [1], respectively) and a given compound using equation [1]

$$\delta_H = \frac{1}{3}Tr\sigma_{H,TMS} - \frac{1}{3}Tr\sigma_H \quad (1)$$

where *Tr* is the trace of matrix, since σ is the tensor with nine elements. The distribution functions of the δ_H values (Fig. S8) were calculated using a simple equation [2]

$$f(\delta_{\rm H}) = (2\pi\sigma^2)^{-0.5}\sum_j \exp[-(\delta_j - \delta_{\rm H})^2 / 2\sigma^2] \quad (2)$$

where *j* is a number of H atom, σ^2 is the distribution dispersion, and δ_j is the calculated value of the *j*-th H atom. Large structures (Fig. S9) were calculated using the PM7 method (MOPAC 2016 package with GPU/CUDA) [2,3]. To calculate the $f(\delta_H)$ functions using the PM7 results, a calibration function was used to describe the dependence between atomic charges q_H (PM7) and the δ_H values (GIAO/ωB97X–D/cc–pVDZ) for water clusters (δ_H = –27.38435372 + 83.67491184×q_H). This function was used to calculate the ^{1}H NMR spectra of water clusters bound to PMS. Visualization of the calculated structures was carried out using ChemCraft [4] or Avogadro 2 (v. 1.91) [5] program suits.

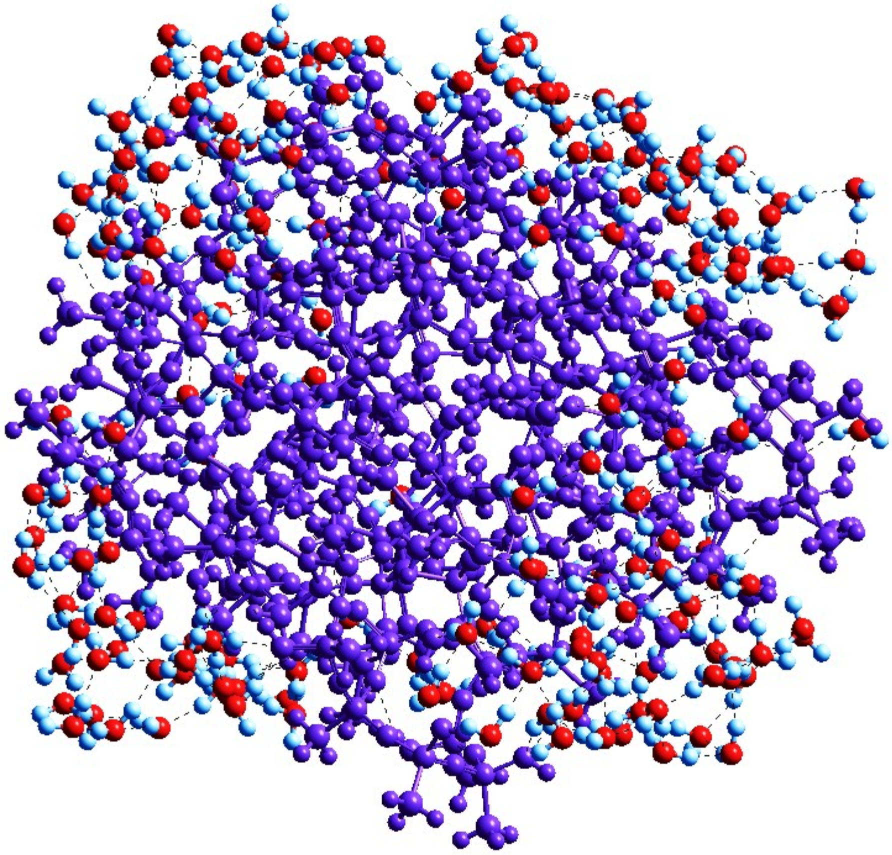

Figure S8. Model of hydrated PMS nanoparticle (PM7 method).

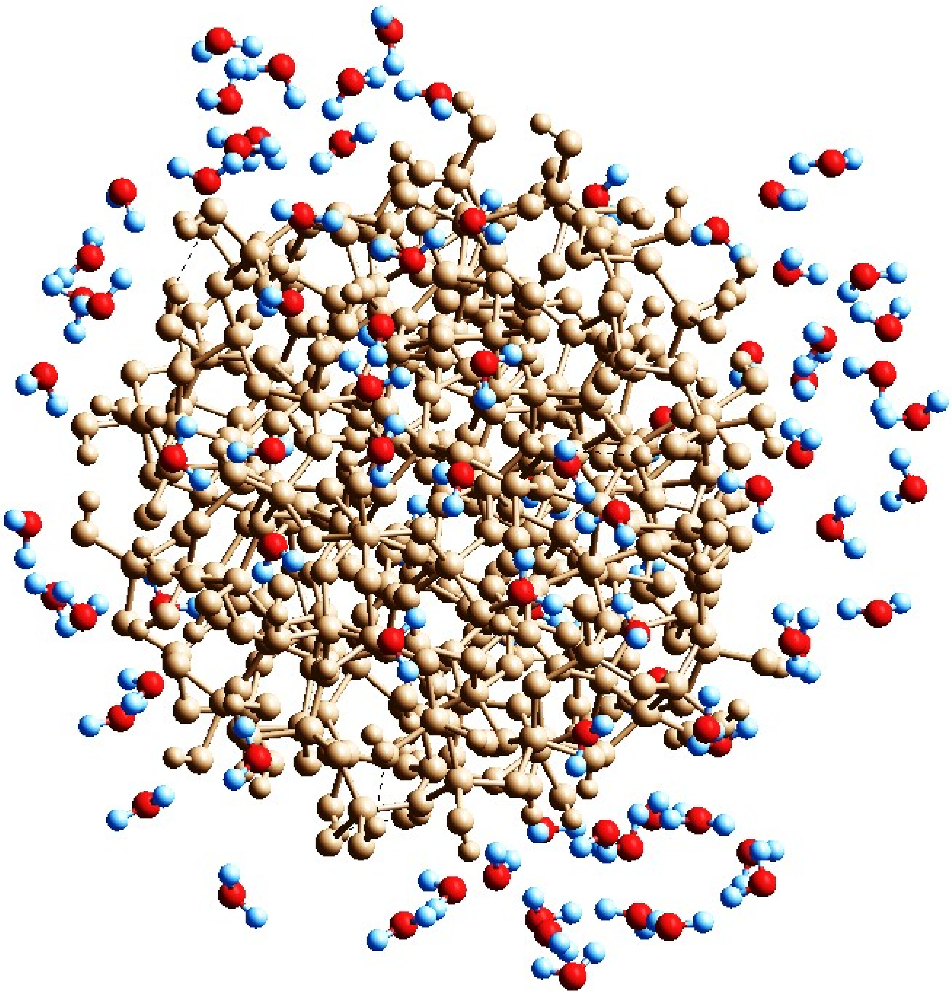

Figure. S9. Model of hydrated silica nanoparticle (PM7 method).

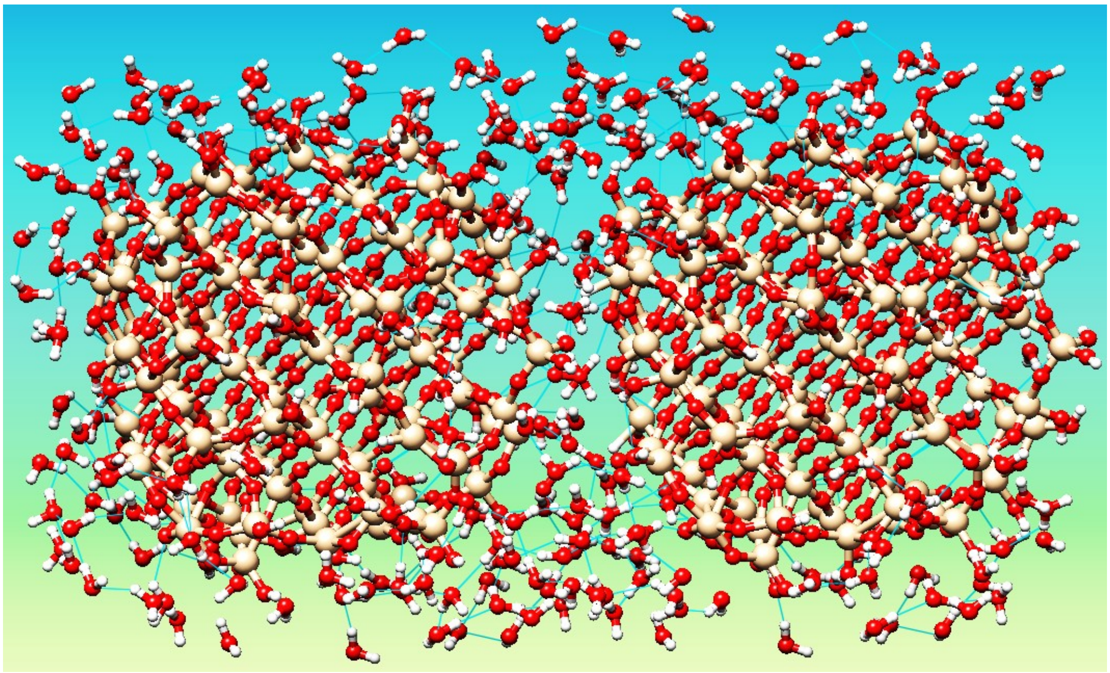

Figure S10. Model of two hydrated silica nanoparticles as a simple aggregate (PM7 method).

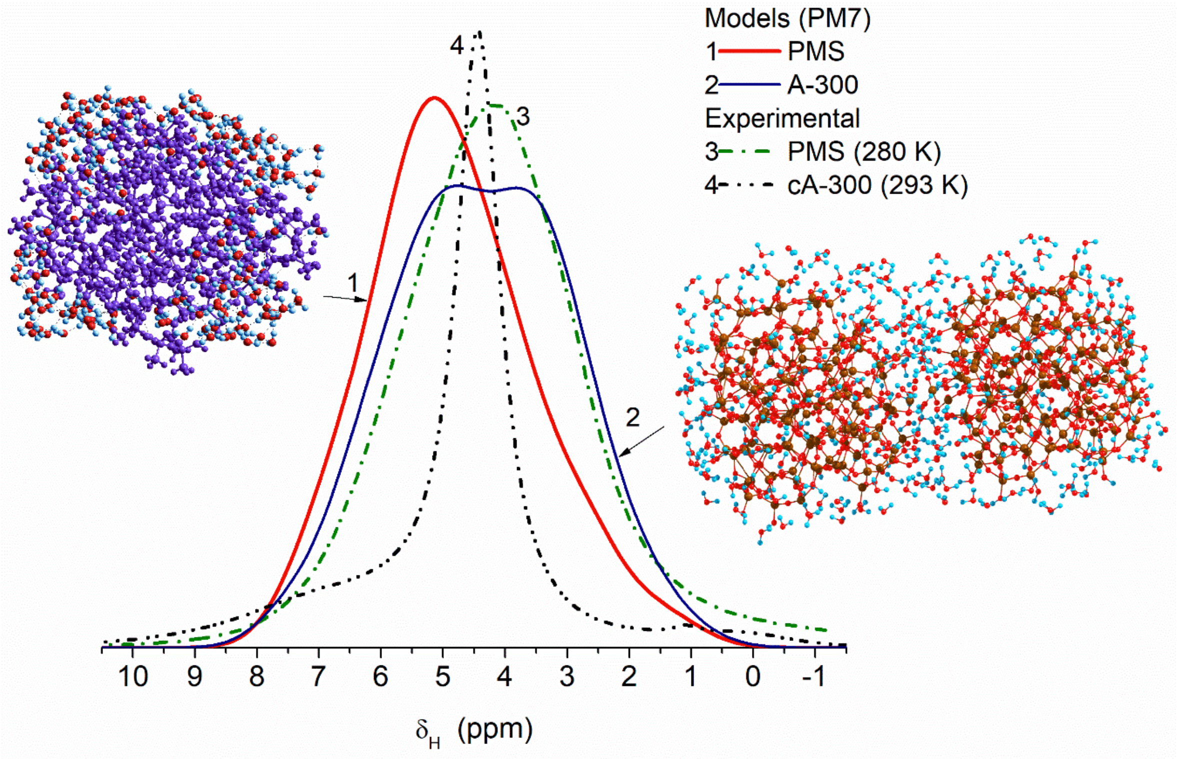

Figure S11. Model ^{1}H NMR spectra (PM7 + correlation function) of hydrated PMS (curve 1) and two nanosilica particles (curve 2); and experimental ^{1}H NMR spectra of static PMS sample (curve 3) and nanosilica (curve 4, ^{1}H MAS NMR).

References

[1] Frisch, M.J.; Trucks, G.W.; Schlegel, H.B.; Scuseria, G.E.; Robb, M.A.; Cheeseman, J.R.; Scalmani, G.; Barone, V.; Mennucci, B.; Petersson, G.A.; Nakatsuji, H. and so on. Gaussian 09, Revision D.01, Gaussian, Inc., Wallingford CT, 2013.

[2] Stewart, J.J.P. MOPAC2016, Stewart Computational Chemistry, web: HTTP://OpenMOPAC.net. Oct. 6, 2017.

[3] Stewart, J.J.P. Optimization of parameters for semiempirical methods VI: more modifications to the NDDO approximations and re-optimization of parameters, *J. Mol. Mod*. **2013**, *19*, 1–32.

[4] Zhurko, G.A.; Zhurko, D.A. Chemcraft (version 1.8, build b536a), 2017, http://www.chemcraftprog.com.

[5] Hanwell, M.D.; Curtis, D.E. Lonie, D.C.; Vandermeersch, T.; Zurek, E.; Hutchison, G.R. Avogadro: an advanced semantic chemical editor, visualization, and analysis platform *J. Chem. Inf.* **2012** *4* 1–17. https://doi.org/10.1186/1758-2946-4-17

© 2019 by the authors. Submitted for possible open access publication under the terms and conditions of the Creative Commons Attribution (CC BY) license (http://creativecommons.org/licenses/by/4.0/).
